# DeepSP: Deep learning-based spatial properties to predict monoclonal antibody stability

**DOI:** 10.1016/j.csbj.2024.05.029

**Published:** 2024-05-18

**Authors:** Lateefat Kalejaye, I-En Wu, Taylor Terry, Pin-Kuang Lai

**Affiliations:** Department of Chemical Engineering and Materials Science, Stevens Institute of Technology, Hoboken 07030, NJ, United States

**Keywords:** Monoclonal antibody, Antibody stability, Molecular dynamics simulation, Spatial charge map, Spatial aggregation propensity, Deep learning

## Abstract

Therapeutic antibody development faces challenges due to high viscosities and aggregation tendencies. The spatial charge map (SCM) and spatial aggregation propensity (SAP) are computational techniques that aid in predicting viscosity and aggregation, respectively. These methods rely on structural data derived from molecular dynamics (MD) simulations, which are computationally demanding. DeepSCM, a deep learning surrogate model based on sequence information to predict SCM, was recently developed to screen high-concentration antibody viscosity. This study further utilized a dataset of 20,530 antibody sequences to train a convolutional neural network deep learning surrogate model called Deep Spatial Properties (DeepSP). DeepSP directly predicts SAP and SCM scores in different domains of antibody variable regions based solely on their sequences without performing MD simulations. The linear correlation coefficient between DeepSP scores and MD-derived scores for 30 properties achieved values between 0.76 and 0.96 with an average of 0.87. DeepSP descriptors were employed as features to build machine learning models to predict the aggregation rate of 21 antibodies, and the performance is similar to the results obtained from the previous study using MD simulations. This result demonstrates that the DeepSP approach significantly reduces the computational time required compared to MD simulations. The DeepSP model enables the rapid generation of 30 structural properties that can also be used as features in other research to train machine learning models for predicting various antibody stability using sequences only. DeepSP is freely available as an online tool via https://deepspwebapp.onrender.com and the codes and parameters are freely available at https://github.com/Lailabcode/DeepSP.

## Highlights

• Deep learning applied to develop a surrogate model (DeepSP) to predict 30 spatial properties of monoclonal antibodies.
• These models achieved an average linear correlation of 0.87, between the actual MD simulations and DeepSP predicted scores.
• DeepSP was used to predict aggregation rates of mAbs with a strong correlation of 0.97, and LOOCV correlation of 0.75.
• DeepSP can generate antibody-specific features to train other machine-learning models to predict antibody stability.

## Graphical Abstract

**Figure:**
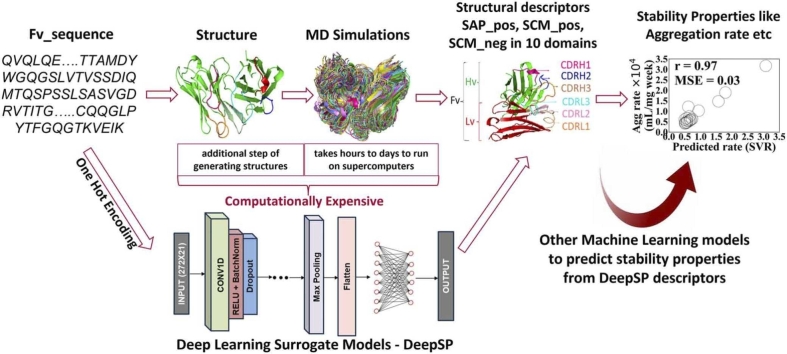

## 1 Introduction

Highly concentrated antibody solutions often exhibit high viscosities [1], aggregation tendencies [2], [3], and various forms of instability, posing significant challenges in antibody-drug development, manufacturing, and administration. Subcutaneous administration requires low-volume and high-concentration formulations [4], [5], [6], [7]. With the increasing desire to improve patient convenience and compliance with monoclonal antibodies (mAbs) by moving away from intravenous and towards subcutaneous mode of administration [8], [9], [10], solutions must be developed to overcome the challenges faced when formulating highly concentrated antibody drugs. The antibody sequence is critical for antibody engineering and acts as a key determinant for high viscosity [1], and other instability issues of highly concentrated solutions. Therefore, developing a sequence-based model that can be used to identify problematic antibodies is desired.

Agrawal et al. [1] developed the spatial charge map (SCM) as a computational tool via molecular dynamics (MD) simulation that can be used for antibody screening to effectively differentiate low or high viscosity antibodies. Chennamsetty et al. [2] developed the spatial aggregation propensity (SAP) as a computational tool via MD simulation that can be used to identify the location and size of aggregation-prone regions and allows target mutations of those regions to engineer antibodies for improving stability. In addition, coarse-grained (CG) models have been implemented in different studies [11], [12], [13], [14], [15] to help screen antibody viscosity and other developability issues. However, these methods are computationally costly and require structural information, which is a significant application bottleneck.

In recent years, machine learning techniques have been adopted in predicting high concentration antibody stability. Lai et al. [16] used machine learning to determine the molecular descriptors responsible for the viscosity behavior of concentrated therapeutic antibodies. The study used 27 FDA-approved antibodies and utilized features based on their charge, hydrophobicity, and hydrophilicity properties. In addition, Lai et al. [17] used machine learning to predict aggregation rates of concentrated therapeutic antibodies. This study utilized 21 high-concentration therapeutic antibody with experimental aggregation rates, obtained SAP and SCM scores from MD simulations across different domains of antibodies as features and employed the feature selection method to select the best four-feature combinations. Moreover, Lai et al. [18] used machine learning to predict antibody aggregation and viscosity at high concentrations (150 mg/mL). This study utilized 20 preclinical and clinical-stage antibodies. Despite the success of these machine-learning models, the features need to be calculated from time-consuming MD simulations.

Deep Learning is a subset of machine learning that consists of many multi-layer neural networks with many hidden units [19], [20]. The common architectures include artificial neural networks (ANN), convolutional neural networks (CNN), and recurrent neural networks (RNN). Unlike traditional machine learning, deep learning can learn features by itself. Deep learning has been adopted in previous studies over the years to study and predict different antibody properties [21], structures [22], [23], [24], ability to bind to target antigen [25], specific B-cell epitope [26], [27], and apparent solubility [28]. Rai et.al [29] used deep learning to predict antibody viscosity at high concentrations using the electrostatic potential surface of the antibody variable region (Fv) as input, which still requires structural information. Lai [30] used deep learning to develop a convolutional neural network surrogate model, DeepSCM, which requires only sequence information to predict the SCM score of antibodies in the entire Fv which can then be used to predict high concentration antibody viscosity. However, DeepSCM only accounts for the surface charges of the Fv region and its predictive capability could further be improved by including other surface descriptors across the different regions. Studies have shown that both charge (obtainable from SCM), solvent-accessible surface area, and hydrophobicity (obtainable from SAP) are key descriptors influencing the aggregation rates and viscosity of monoclonal antibodies (mAbs) [16], [17], [18]. In light of this, a promising avenue for advancing the prediction accuracy of antibody stability during early-stage drug discovery and development involves the creation of an antibody-specific sequence-based tool. Such a tool would comprehensively capture both charge and hydrophobicity, offering a more holistic approach to predicting antibody behavior.

In this study, we applied deep learning to develop DeepSP, a collection of different surrogate models that can be used to predict average dynamic SCM and SAP scores in different domains of an antibody not just the entire variable region with a much larger and diverse datasets (N = 20530) solely based on the antibody sequences, thereby accelerating MD simulations and providing a more comprehensive and holistic model for predicting antibody behavior. The sequences used for model training were obtained from the Observed Antibody Space (OAS) database [31]. First, we performed MD simulations to calculate the dynamic average and standard deviation of SAP_positive (SAP_pos), SCM_negative (SCM_neg) and SCM_positive (SCM_pos) scores in the CDRH1, CDRH2, CDRH3, CDRL1, CDRL2, CDRL3, CDR, Hv, Lv and Fv regions of these antibodies. This process yielded a total of 30 structural properties, as summarized in Table 1. We then trained a deep learning surrogate model – DeepSP, using these MD-derived averages as outputs and the preprocessed antibody sequences as inputs for model training. The relative standard deviation was utilized to quantify the error of uncertainty in the prediction of the average scores. The linear correlation coefficient of the DeepSP scores and MD-derived scores for these properties achieved values between 0.76 and 0.96 with an average of 0.87 on test set (N = 2053).

**Table 1:** List of mAb properties and domains in DeepSP model. The properties are calculated with an in-house program.

mAb Properties	Domains
Spatial aggregation propensity (SAP_pos) Spatial negative charge map (SCM_neg) Spatial positive charge map (SCM_pos)	CDRH1 CDRH2 CDRH3 CDRL1 CDRL2 CDRL3 CDR Hv Lv Fv

To further validate the performance of DeepSP, we utilized a dataset comprising aggregation rates of 21 high-concentration (150 mg/mL) mAbs obtained from a previous study [17]. In this study, we employed a similar approach to the original study, using machine learning models to predict antibody aggregation rates. However, instead of using MD simulations to generate features, we utilized the DeepSP model to predict 30 structural properties of 21 antibodies, which we used as inputs (features) to train various machine learning models. We observed remarkable results, with a high correlation coefficient (R = 0.97) and low mean squared error (MSE = 0.03) between the experimental and predicted aggregation rates. Leave-one-out cross-validation (LOOCV) yielded a correlation coefficient (R = 0.75) and MSE value (MSE = 0.18). This is similar to the results obtained from the previous study that used MD simulations to generate the same features to train a machine learning model to predict their aggregation rates achieving R= 0.94 and MSE = 0.08, with a LOOCV validation yielding R = 0.77 and MSE = 0.22.

These **DeepSP** features can also serve as input in other research to train other machine learning or deep learning models to predict other desired stability properties of the antibodies with known and available sequences. By implementing this deep learning model during antibody screening or engineering processes, it becomes possible to identify antibodies that may have stability issues, allowing for targeted re-engineering or removal from the antibody panel.

## 2 Methods

### 2.1 Antibody sequence datasets and preprocessing

Antibody sequences were retrieved from the Observed Antibody Space – OAS [31], Duplicated antibody sequences, and those with unpaired Fv regions were removed from the dataset. The length of these antibody sequences varies and was therefore annotated with the IMGT numbering scheme using ANARCI [32] to ensure the same input size was achieved for deep learning algorithms. The heavy chain and light chain variable regions ranged from H1 to H128 and L1 to L127, respectively, with gaps filled by dashes. The maximum length allowed on the CDRH3 region [H105-H117] was 30 because the majority of the antibody sequences in the dataset used for training, do not have more than 30 residues in the CDRH3 region. Sequences with insertions on other CDR or framework regions were removed from the dataset. Furthermore, sequences that do not have exactly two cysteine residues at positions 23 and 104 in the heavy and light chains were removed. The sequences of the heavy chain and light chain were aligned and preprocessed separately at this stage. Subsequently, homology models were generated using these aligned sequences. Any sequences that failed to generate homology models for the Fv regions were excluded from further analysis. This approach was adapted from a previous study [30]. Overall, these steps resulted in a total of 23,520 antibody Fv sequences being retained for subsequent analysis.

### 2.2 Computational modeling of mAbs and molecular dynamics simulations

The homology models of the Fv regions were generated by ABodyBuilder-ML [33] using the heavy chain and light chain separately as input. IMGT numbering was used to annotate the final models. The Fv structures were generated as pdb files (a sample structure is provided as Fv.pdb in Supporting Information). The missing residues at the C and N terminals were generated using the target_numbering file of the original homology model from AbodyBuilder-ML and appended. The cysteine residues were joined to form a disulfide bond both on the heavy and light chains, and then the modified pdb file, which contains the atomic coordinates, was generated. The antibody Fv-structure was immersed in explicit solvent using the TIP3P water model [34]. The simulation setup involved placing a single antibody Fv structure in a water box extending 12 Å beyond the protein surface using VMD [35]. The system was neutralized with counterions. Histidine residues were protonated at pH 6 using PROPKA [36]. pH 6 was chosen for this study as it is the optimal stability point for formulating most mAbs [37], [38]. Specifically, only histidine residues were protonated at this pH because their pKa value matches the pH, allowing for a transition between neutral and charged states [38], unlike other residues have relatively stable positive or negative charges at pH 6, because their pKa values are far from the pH values. The electrostatic interactions were treated with the PME method. Van der Waals interactions were calculated with a switching distance of 10 Å and a cutoff of 12 Å [39]. Following energy minimization, the system was gradually heated up from 100 K to 300 K at an interval of 5 K over 200 ps. The heavy atoms were constrained with a harmonic constraint energy function scaled at 2.5 kcal/Å^2^. Subsequently, the constraints were incrementally relaxed by adjusting the scaling factor to 2.0, 1.5, 1.0, and 0.5 kcal/Å^2^ over an 80 ps period. A 1 fs time step was employed during the heating and relaxation phases. The equilibrium and production were performed at 300 K and 1 atm in the NPT ensemble using NAMD software [40] and the CHARMM36m force field [41], [42]. The simulation was run with 10 ns equilibrium and 10 ns production run, and the integration time step was set to 2 fs by applying rigid bond constraints to hydrogen-containing bonds. 20530 antibody sequences were retained after the MD simulation stage. We proceeded to calculate the dynamic averages of SCM and SAP scores of the remaining antibodies as described in the next section. It is worth noting that structural features can be obtained either from a single snapshot or from the average of multiple structures. As demonstrated by a previous study, ensemble averages derived from dynamic simulations provide a more accurate representation of SAP and SCM scores compared to static scores obtained from individual 3D structures (single snapshot) [17]. This highlights the significance of utilizing dynamic average values for structural features. We leveraged the combined computational resources of three clusters (Expanse, Anvil, and Summit), utilizing multiple GPUs to accelerate our simulations, which took around six months to finish.

### 2.3 Calculation of spatial charge map and spatial aggregation propensity scores

The spatial charge map (SCM) is a score that was developed to differentiate low or high antibody viscosity in high concentrated solutions. The calculation of SCM scores follows previous work [1]. Briefly, the atomic SCM value has the following form.

(1) $$\mathrm{SC}M_{i}=<\underset{\left(\mathrm{exposed}\;\mathrm{residues}\;<\;10Å\right),j}{\sum }\left(q_{j}\right)>$$

where < > indicates ensemble average from MD simulations. The atomic SCM value ($\mathrm{SC}M_{i}$) is the summation of all the partial charges (*q*_*j*_) on the surrounding atom j, which are within 10 Å of atom i that belongs to exposed residues. The exposed residues are considered if the sum of the side chain solvent accessible area is ≥ 10 ${Å}^{2}$**.** The SCM score in different regions is then expressed as:

(2) $$\text{SCM\_neg}\text{ score}=\left|\sum_{\mathrm{domain}}\text{SC}{\text{M}}_{i}\times H\left(-\mathrm{SC}M_{i}\right)\right|$$

(3) $$\text{SCM\_pos}\text{ score}=\left|\sum_{\mathrm{domain}}\text{SC}{\text{M}}_{i}\times H\left(\mathrm{SC}M_{i}\right)\right|$$

where domain refers to CDRH1, CDRH2, CDRH3, CDRL1, CDRL2, CDRL3, CDR, Hv, Lv, and Fv, H is the Heaviside function, and $\left|.\right|$ is the absolute value function.

The spatial aggregation propensity (SAP) is a tool used to identify the location and size of aggregation-prone regions in antibodies. The calculation of SAP follows previous work [2]. The atomic SAP value is calculated as

(4) $${\mathrm{SAP}}_{i}=\sum_{\begin{array}{c} \mathrm{Simulation}\\ \mathrm{Average} \end{array}}\left\{\sum_{\begin{array}{c} \mathrm{residue}\\ \mathrm{with}\;\;\mathrm{at}\;\;\mathrm{least}\\ \mathrm{one}\;\;\mathrm{side}\;\;\mathrm{chain}\\ \mathrm{atom}\;\;\mathrm{within}\;\;R\\ \mathrm{from}\;\;\mathrm{atom},i. \end{array}}\left(\frac{\mathrm{SAA}\;\mathrm{of}\;\;\mathrm{side}\;\;\mathrm{chain}\;\;\mathrm{atoms}\;\;\mathrm{within}\;\;\mathrm{radius}\;\;R}{\mathrm{SAA}\;\;\mathrm{of}\;\;\mathrm{side}\;\;\mathrm{chain}\;\;\mathrm{atoms}\;\;\mathrm{of}\;\;\mathrm{fully}\;\;\mathrm{exposed}\;\;\mathrm{residue}}*\mathrm{Residue}\;\mathrm{Hydrophobicity}\;\right)\right\}$$

(5) $$\text{SAP\_score}=\left|\sum_{\mathrm{domain}}\text{SAP}\times H\left({\mathrm{SAP}}_{i}\right)\right|$$

The SAP values in different regions, CDRH1, CDRH2, CDRH3, CDRL1, CDRL2, CDRL3, CDR, Hv, Lv and Fv, were also obtained.

It is noted that the traditional SCM and SAP are based on the atomic SCM and SAP. For traditional SCM, all the atomic SCM scores are added for the entire variable regions. For traditional SAP, all the atomic SAP scores are added for each residue. This work expands the SCM and SAP calculation to various regions of antibodies.

### 2.4 Development of DeepSP using deep learning models

One approach commonly used to represent proteins in machine learning is the one-hot encoding [43], [44]. In this study, the heavy chain and light chain of the antibody sequence were concatenated and encoded as a single binary vector of length 21, representing the 20 amino acids and one gap. This combined sequence was used as input to the deep learning models, with each vector consisting of 20 zeros and a single one, where the position of the one indicates the specific amino acid residue at that position in the protein sequence. This encoding approach is often used with advanced machine learning algorithms such as convolutional neural networks [45]. Deep learning models were developed in Python 3.9.13 utilizing scikit-learn v1.0.2 [46] for the train_test_split function and Keras v2.11.0 [47] sequential model as a wrapper for TensorFlow v2.11.0 [48]. The CNN architecture employed in this study consisted of three convolutional layers, each integrated with batch normalization and dropout layers, followed by a pooling layer, flattening operation, and a densely connected layer with a single output layer. The number of convolutional layers was manually varied between 3, 4, and 5 but it was found that increasing the number of convolutional layers did not improve model performance. Consequently, the final model architecture utilized three convolutional layers.

Hyperparameter optimization was performed using the Keras Tuner [49] library with three different optimization techniques - Hyperband, Random Search, and Bayesian Optimization techniques [50] to efficiently explore the hyperparameter space and identify the best-performing configurations for the neural network model. Various combinations of hyperparameters were explored, including the number of filters (ranging from 16 to 128 with increments of 16), kernel sizes (selected from [3, 4, 5]), dropout rates (ranging from 0.0 to 0.5 in steps of 0.1), number of units in dense layers (ranging from 32 to 128 with a step of 16), and learning rates (chosen from [1e-2, 5e-3, 1e-3, 1e-4]). The optimal configuration was determined based on the MAE values of the best validation model. The dataset for regression was divided into training (65%), validation (25%), and test sets (10%). The best hyperparameters obtained from keras tuner were used to train the model over 50 epochs with a batch size of 32 and the Adam optimizer, a popular and efficient stochastic gradient descent algorithm. The best model, which is the model with the minimum validation loss was saved using Model Checkpoint from keras.callbacks, and the CNN architecture and weights were saved in JSON and HDF5 formats, respectively. The activation function used for the CNN model was ReLU. Other activation methods that can be considered (though not evaluated in this study) are LeakyRelu, Swish.

In our study, two different approaches were employed to predict the spatial properties in antibodies using the methods described above. First, we trained individual CNN models for each of the 30 properties, resulting in a total of 30 models in our DeepSP collection. Second, we trained three models, each predicting a property (SAP_pos, SCM_pos, or SCM_neg) across all 10 regions of the antibodies, resulting in 10 outputs per model. For instance, the SAP_pos model can predict properties across different antibody regions, such as SAP_pos_CDRH1, SAP_pos_CDRH2, and so on. No significant differences were observed in the predictions after comparing the outcomes of these two approaches. Detailed information on best hyperparameters and model performance for both approaches can be found in Tables S1-S4 in the Supporting Information. We decided to adopt the latter approach for the rest of the project to save the time and computing resources needed to train and tune three (3) models instead of thirty (30).

### 2.5 Machine learning feature selection and modeling to predict aggregation rate using DeepSP features

To validate the performance of the DeepSP model established in this study, we utilized a dataset comprising aggregation rates of 21 high-concentration (150 mg/mL) mAbs obtained from previous research [17]. DeepSP was used to generate 30 structural properties as features in machine learning model training. Given the limited dataset size, the risk of overfitting [51] arises when dealing with numerous features. Therefore, we applied the Exhaustive Feature Selector (EFS) tool from mlxtend library [52] in conjunction with various regression algorithms for feature selection. We iteratively assessed different feature subsets based on the negative mean squared error as the scoring metric, varying feature counts, and cross-validation folds. Subsequently, we computed the mean MSE for specific feature subsets identified by the EFS. For each subset, MSE was computed using different regression models using a repeated k-fold cross-validation method. Finally, we collected all subset details and their associated averaged MSE values, selecting the feature combination with the smallest MSE value to train the machine learning model.

The machine learning algorithms from the scikit-learn library [46] used are linear regression (LR), k-nearest neighbors regressor (KNN), support vector regressor (SVR) and random forest regressor (RFR). After selecting the best feature combinations obtained from the exhaustive feature combination, each machine learning model was trained and tuned to obtain the optimal hyperparameter that will give the best model. For KNN, we varied the number of neighbors from 2 to 8, for SVR, we tuned the parameters C (ranging from 5.0 to 15.0) and ε (ranging from 0.1 to 0.5), while for RFR, we adjusted the max_depth parameter (ranging from 2 to 6). We then evaluated each model's performance by comparing the correlation coefficients (r) and MSE between the experimental and predicted data. The model that exhibited the highest correlation coefficients and the lowest MSE was selected as our final machine-learning model. To verify the reliability of our models, we implemented LOOCV, a commonly used technique in machine learning and statistics for model performance assessment, particularly in situations with limited data. While tuning the parameters, we concurrently created a validation model using LOOCV with the same set of parameters. Although the correlation coefficients and mean square error of the validation model exhibited slightly worse performace compared to the initial model, we established a threshold. If the correlation coefficients did not decrease by more than 0.3, we considered the model as yielding reliable results.

## 3 Results and discussions

### 3.1 Antibody sequence dataset and statistical analysis

The antibody variable region paired sequences (30,523) were retrieved from OAS [31]. The preprocessing steps (detailed in the Materials and Methods section), which includes filtering out sequences based on some criteria such as complementarity determining region (CDR) length, the number of cysteine residues, and insertion yielded 25320 sequences and after removing the ones that failed during MD simulations, 20530 antibody Fv sequences were left for this study.

**Fig. 1:**
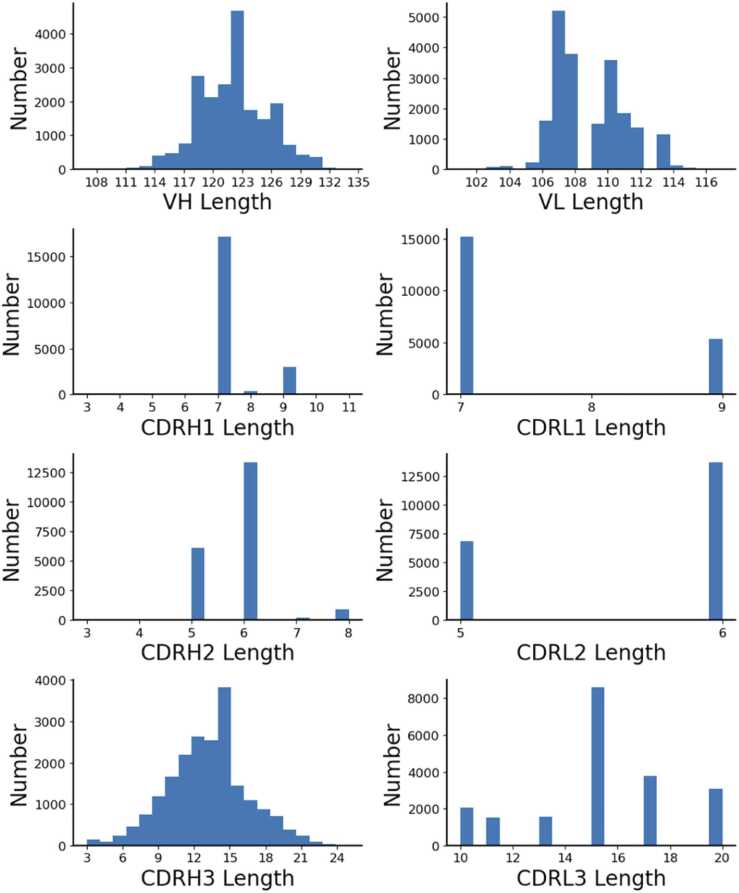
Distribution of VH, VL, CDRH1, CDRH2, CDRH3, CDRL1, CDRL2, and CDRL3 lengths of the 20530 Fv sequences in this study. The CDR regions are based on the Chothia definition.

Fig. 1 shows the length distribution of different antibody regions in the dataset. The VH length and VL length were approximately normally distributed, centered at 122 and 108 respectively. The first complementarity determining region of the heavy chain (CDRH1) length had the highest peak at 7 which constitute about 85% of the data set, and the rest had length of 8–9. The first complementarity determining region of the light chain (CDRL1) length had the highest peak at 7 which constitutes about 70% of the dataset and the rest had length of 9. For the second complementarity determining region of the heavy chain (CDRH2) length, the highest peak was at 6, and the second-highest peak was at 5 and the rest has 7 or 8. The second complementary determining region of the light chain (CDRL2) length had the highest peak at 6 and the rest has length of 5. For the third complementarity determining region of the light chain (CDRL3) length, the highest peak was at 15. The third complementary determining region of the heavy chain (CDRH3) length had a wide distribution centered at 13.

CNN models require the input to have a fixed size, however, our antibody sequences have variable lengths. To address the variable length issue, we adopted the Chothia numbering scheme [53] to annotate the heavy and light chain variable regions. This choice was made over IMGT due to Chothia's focus on conserved CDRs, enabling better alignment and representation of antibody functionality. With Chothia, we ensured the CDRs were accurately captured, allowing for precise analysis and modeling of antibody properties. Gaps were padded with dashes, resulting in fixed lengths of 145 and 127 for the heavy and light chain variable regions, respectively.

### 3.2 MD simulations, SCM and SAP calculation of the antibody in the dataset

**Fig. 2:**
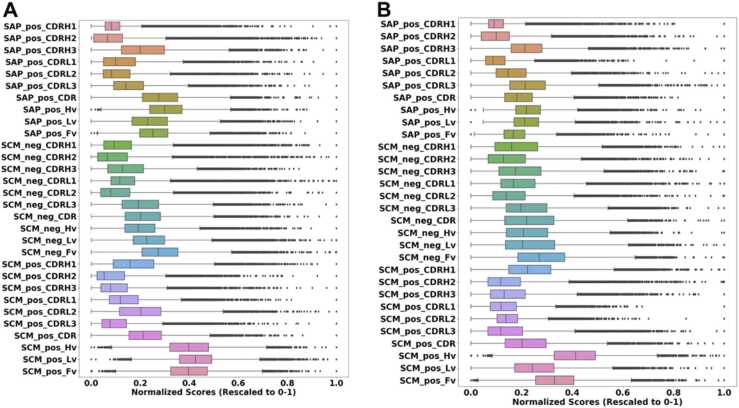
Box-and-Whisker plot illustrating the normalized (rescaled to 0 −1) A) average B) standard deviation score distribution for all 30 properties obtained from MD simulations.

The homology models of the antibody variable regions were constructed and prepared to perform MD simulations. To confirm the appropriate equilibrium and production run time, we conducted a 10 ns equilibrium run and a 50 ns production run for an antibody in our dataset to determine the optimal production run time for stabilizing the desired properties. Fig. S1 shows the time trajectory plot of the SAP_pos, SCM_neg, SCM_pos scores in the 10 regions of an antibody considered in this study. The scores fluctuated around the mean, and the mean converged and stabilized after 10 ns production run, hence 10 ns equilibrium run, and 10 ns production run was maintained for the other antibodies. Unlike in full-length antibodies, which demand extended simulation times for convergence and stability, single-variable region simulations achieve quicker equilibration which makes them more suitable for high-throughput computing in large antibody datasets. 20530 antibody sequences were retained after MD simulation. The final annotated CSV file, provided as Supporting Information (annotated_oas_data.csv), contains all included antibody sequences retrieved from the OAS database, along with reasons for excluding those that did not meet the criteria up to this stage. The SAP_pos, SCM_pos and SCM_neg scores were calculated in the CDRH1, CDRH2, CDRH3, CDRL1, CDRL2, CDRL3, CDR, Hv, Lv, and Fv regions by the ensemble averages over 10 ns. Fig. 2 shows the box-and-whisker of the normalized average and standard deviation score distribution for all the 30 spatial properties as obtained from MD simulation. Table S5 summarizes the analysis of the 30 spatial properties.

### 3.3 CNN model training and optimization for the DeepSP model

CNN model was chosen for model development in this study as it has been shown to perform better than other deep learning models like ANN and RNN for predicting antibody binders [54]. The ratio for training/validation/test split was 65:25:10. The architecture and parameters were optimized by tuning hyperparameters using keras tuner (as detailed in the Materials and Methods section).

**Fig. 3:**
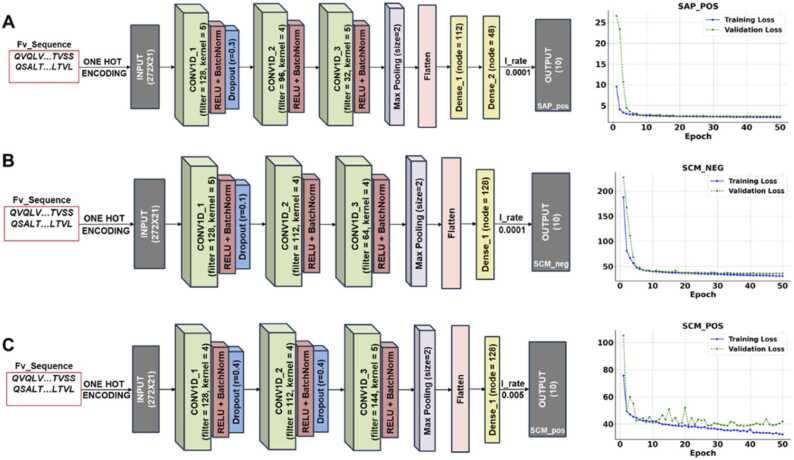
Illustration of CNN model architecture with the training and validation loss over number of epochs for A) SAP_pos model B) SCM_neg model C) SCM_pos model, contained in DeepSP surrogate model developed in this study.

Fig. 3 shows the CNN architecture of the three models. Each model had an input shape of (272, 21). The number of columns is the sum of heavy chain variable region length (145) and light chain variable region length (127). The rows came from one-hot encoding, including 20 amino acids and one gap. The input layer was connected to a 1D CNN layer using the activation function of the rectified linear unit (ReLU). Fig. 3 illustrates the architecture, and also displays the training and validation loss curves over the training epochs for all three models. While the training and validation loss generally converge, there is noticeable divergence in the case of SCM_pos after 20 epochs, which indicates impeding overfitting with increasing number of epochs. However, we used model checkpoint from keras callback to monitor the model performance and implemented the model with the minimum validation loss, which was determined by the mean absolute error (MAE) metric.

**Fig. 4:**
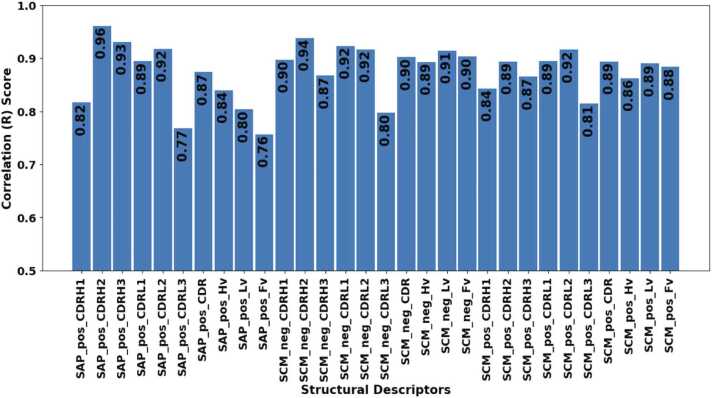
Bar plot illustrating the correlation between the predicted and actual score of all 30 spatial properties.

Table S3 shows the best and optimal hyperparameter combination generated from keras tuner for each of the three models based on the minimum mean absolute error (MAE). Table S4 detailed the mean score, baseline mean absolute error, validation loss, mean absolute error and correlation between actual and predicted scores for each property. Fig. 4 shows the correlation between the predicted and actual scores for all properties. A minimum correlation of 76% and maximum correlation of 97%, and the MAE of all the properties greatly beats the baseline MAE (calculated with mean) as shown in scatter plot illustrated in Fig. S2. The relative standard deviation was obtained by dividing the standard deviation by the actual value and can be found for each property in Fig. S3.

### 3.4 Aggregation rate prediction using DeepSP model as features

In the previous study [17], a machine-learning model was proposed to predict the aggregation rates of 21 mAbs. MD simulation was employed to compute the SAP and SCM in different mAb domains, which served as features for machine learning model training. Here, we used DeepSP developed in this study to predict these features solely from the Fv sequences, as demonstrated in Table 1. This is to validate the ability of DeepSP features to be able to alleviate the computationally expensive MD simulation in generating these features for training machine learning models to predict the aggregation rate of an antibody.

For feature selection, various feature combinations were explored for Linear Regression, Support Vector Regression (SVR), Random Forest (RF), and K-Nearest Neighbors (KNN) models using 4-fold cross-validation. Subsequently, we computed the MSE values for machine learning models built using different feature combinations. These MSE values were used to determine the optimal set of features for subsequent machine learning model training. Table 2 summarizes the MSE values calculated for three and four feature combinations to identify those with the lowest MSE values for subsequent hyperparameter tuning of the models. This method ensures the effective selection of optimal feature combinations during machine learning model training, thereby enhancing their predictive and generalization capabilities. It is noteworthy that the machine learning algorithms frequently selected CDRH3, possibly due to its high sequence diversity in that region.

**Table 2:** Mean squared error (MSE) of the best three-feature and four-feature combinations of the linear regression, support vector, k-nearest neighbors, and random forest regression models for predicting aggregation rates. Hyperparameters are set to the default Scikit-learn parameters.

**Regression Models**	**Three-feature**	**MSE**	**Four-feature**	**MSE**
Linear	SAP_pos_CDRH3 SCM_pos_CDRL3 SCM_neg_CDRH3	0.433	SAP_pos_CDRH3 SCM_pos_CDRL1 SCM_pos_CDR SCM_pos_Hv	0.457
Nearest neighbors (neighbor numbers = 5)	SAP_pos_CDRL3 SCM_pos_CDRH3 SCM_neg_Fv	0.366	SCM_pos_CDRH3 SCM_neg_CDRH2 SCM_neg_Hv SCM_neg_Fv	0.319
Random forest (max_depth = None)	SAP_pos_Hv SCM_pos_CDRH3 SCM_neg_Fv	0.367	SAP_pos_Hv SCM_pos_CDRH3 SCM_pos_Lv SCM_neg_Fv	0.364
Support vector (C = 1.0, ε = 0.1)	SCM_pos_CDRH3 SCM_neg_CDRH2 SCM_neg_Fv	0.307	SCM_pos_CDRH3 SCM_neg_CDRH2 SCM_neg_CDRL2 SCM_neg_Fv	0.301

**Fig. 5:**
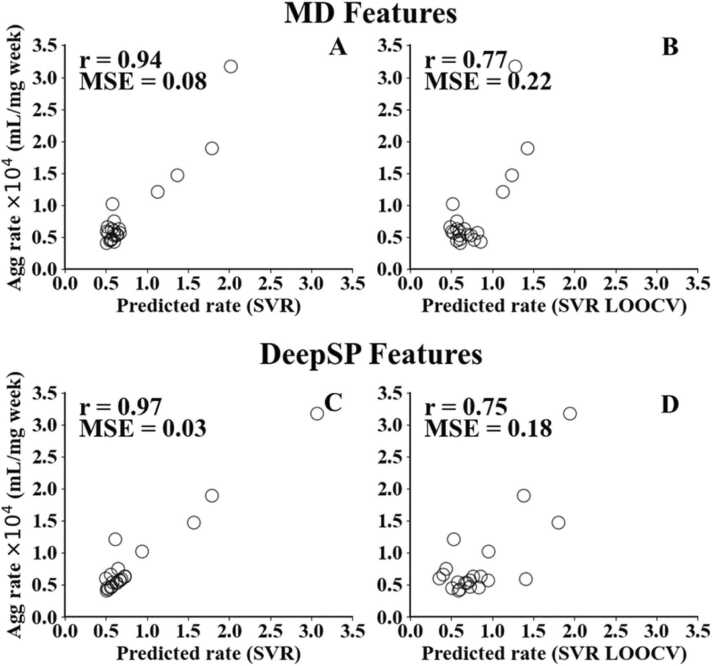
Scatter plot of correlation between predicted and experimental aggregation rate A, B) from previous study where MD simulation features were used C, D) current study where DeepSP (sequence-based) features were used.

After tuning the hyperparameters, Table 3 summarizes the results of the best three-feature or four-feature combinations of Linear Regression, SVR, RF, and KNN models. The SVR model has the highest correlation coefficient of 0.97 and a MSE of 0.03 when comparing the experimental data to the predicted data. (For comparisons with other regression models, refer to Fig. S4.) Following a similar validation approach as the previous study, we employed the leave-one-out-cross-validation (LOOCV) on our limited dataset to validate our training results and ensure its reliability. LOOCV provides a dependable estimate of a model's performance and is particularly valuable for detecting issues like overfitting, especially in scenarios with small datasets where leveraging available information is crucial. Table 3 summarizes the performance of all regression models in comparison to their LOOCV performance which shows that SVR model, evaluated using LOOCV, yielded a correlation coefficient (r) of 0.75 and a MSE of 0.18. While the correlation coefficient exhibited a slight reduction, it remained within acceptable limits. Furthermore, when compared to other regression models, the SVR model outperformed them. These results closely align with the performance obtained in the previous study [17] where features were derived from MD simulations as shown in Fig. 5. This demonstrates the effectiveness of our newly established DeepSP model, which can effectively replace the MD-based methods.

**Table 3:** Performance metrics, correlation coefficients (r) and mean square error (MSE) of different regression models.

**Regression Models**	**Features**	**r (all)**	**r (LOOCV)**	**MSE (all)**	**MSE (LOOCV)**
Linear	SAP_pos_CDRH3 SAP_pos_CDRL3 SCM_neg_CDRH3	0.49	0.14	0.31	0.40
Nearest neighbors (neighbor numbers = 3)	SCM_pos_CDRH3 SCM_neg_CDRH2 SCM_neg_Hv SCM_neg_Fv	0.85	0.64	0.11	0.24
Random forest(max_depth = 6)	SAP_pos_Hv SCM_pos_CDRH3 SCM_pos_Lv SCM_neg_Fv	0.94	0.47	0.05	0.32
Support vector (C = 15.0, ε = 0.1)	SCM_pos_CDRH3 SCM_neg_CDRH2 SCM_neg_Fv	0.97	0.75	0.03	0.18

Due to the limited size of the dataset, it is possible that the machine learning model may select slightly different features if another dataset is employed. However, it is expected that the physical interpretation and meaning of the selected features should remain consistent. For instance, in the previous publication [17] we aimed to compare with, the most essential feature selected was the positive charge in the variable region (SCM_pos_Fv), which aligns with the most important feature selected in our study (SCM_pos_CDRH3). This consistency suggests that positive charge contributes to repulsive interactions on the surface of antibodies that can affect their aggregation rates.

Also, the variability in feature combinations selected by machine learning algorithms could be due to the limited size of the dataset. With a larger dataset, most of the models should be able to capture the same feature combinations. For instance, in a previous study [18], as the dataset expanded from 20 to 47, the machine learning algorithms selected consistent features for high viscosity.

### 3.5 Availability and implementation of the DeepSP model

DeepSP is freely available as an online tool and can be assessed via https://deepspwebapp.onrender.com. The name, heavy chain and light chain of the antibody are to be inputted on the web form page and upon submitting, the DeepSP descriptors are generated and displayed. The source codes and parameters are freely available at https://github.com/Lailabcode/DeepSP, which can also be used to generate descriptors for large antibody sequences at once. The notebook file – DeepSP_predictor.ipynb can be run locally on google colab, which requires only one input which is a csv file that contains the names, heavy chain, and light chain (Fv sequences only) of the antibody whose descriptors are to be generated (see DeepSP_input.csv for sample format). The python file - DeepSP_train.py contains the code that was used for DeepSP training, validation, and testing.

## 4 Conclusion

DeepSP was developed as a surrogate model to accelerate the MD simulation-based tools for calculating SAP and SCM scores in all 6 CDR regions, the entire CDR region, Hv, Lv, and the entire Fv region of an antibody solely from the sequence. It was trained using high-throughput MD simulation results and 1D convolutional neural network architecture. DeepSP, as an antibody-specific model, incorporates features such as charge and hydrophobicity. This makes it a more comprehensive descriptor for antibodies, enhancing its capability to predict and assess antibody stability accurately. DeepSP has been used to predict spatial properties, which served as input or features to an SVR model, trained to predict the aggregation rate of 21 monoclonal antibodies.

DeepSP features can serve as antibody-specific features for training machine learning models for other stability properties such as viscosity (manuscript in preparation) and solubility as well as other desired properties using only Fv sequences. These tools can screen for hundreds of antibody drug candidates within a few seconds. The DeepSP features can also be used to train surrogate models for other biophysical properties from experiments, such as melting temperature, retention time from hydrophobic interaction chromatography, etc. It is important to clarify that the goal of this study is not to directly predict stability properties, as their experimental data is necessary to train machine learning models. The aim of this study is to expedite the MD simulation process for generating antibody-specific descriptors. These descriptors can then be integrated into other machine learning models alongside actual experimental stability properties. Also, this study only evaluated SAP and SCM descriptors, we plan to explore other useful descriptors that can give insight into antibody stability in the future. Deep learning paves a promising way for predicting antibody functions to facilitate drug design. Overall, this tool will facilitate early-stage drug development.

## CRediT authorship contribution statement

**I-En Wu:** Visualization, Software, Methodology. **Lateefat Kalejaye:** Writing – review & editing, Writing – original draft, Visualization, Validation, Software, Methodology, Data curation, Conceptualization. **Pin-Kuang Lai:** Writing – review & editing, Supervision, Methodology, Funding acquisition, Conceptualization. **Taylor Terry:** Data curation.

## Declaration of Competing Interest

There is no conflict of interest.
